# Etymologia: Negri Bodies

**DOI:** 10.3201/eid2309.ET2309

**Published:** 2017-09

**Authors:** Ronnie Henry, Frederick A. Murphy

**Keywords:** etymologia, Negri bodies, rabies, rabies virus, viruses, cytoplasmic inclusions, neurons, diagnosis, Adelchi Negri

## Negri [negʹrē] Bodies

**Figure 1:**
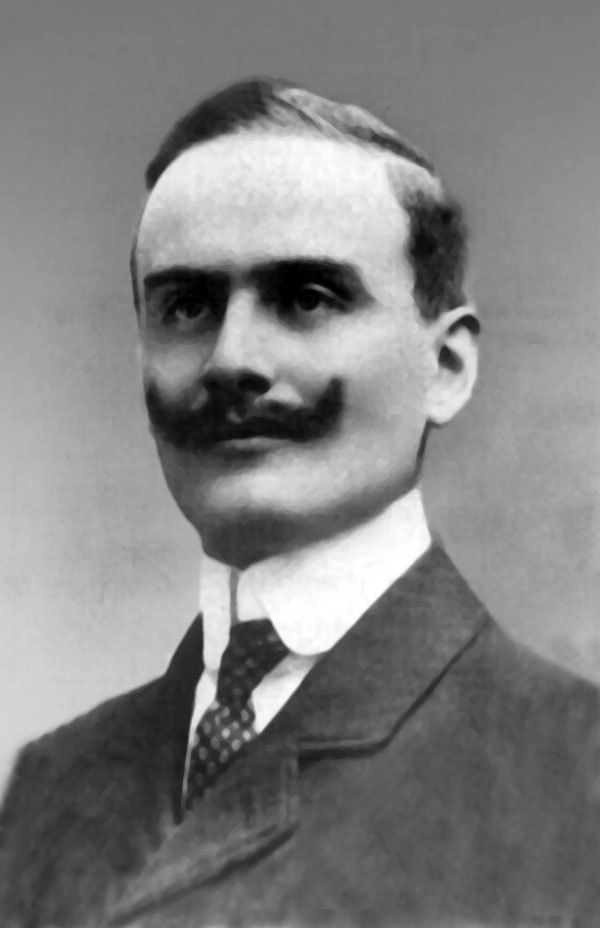
Neuron showing a cytoplasmic inclusion body (Negri body, arrow). Courtesy Frederick A. Murphy.

**Figure 2:**
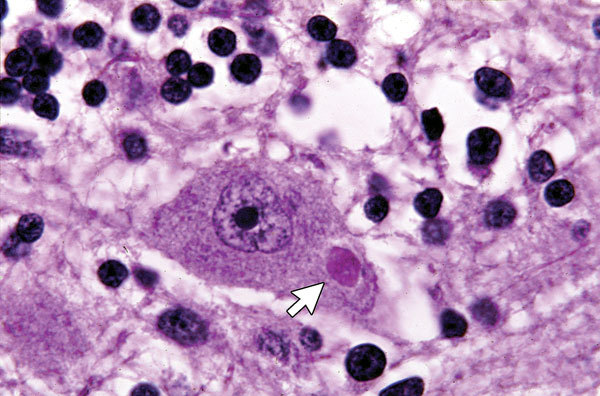
Adelchi Negri 1876–1912, Courtesy Frederick A. Murphy

Negri bodies (Figure 1) are cytoplasmic inclusions in neurons that are composed of rabies virus proteins and RNA. Adelchi Negri (Figure 2), an assistant pathologist working in the laboratory of Camillo Golgi, observed these inclusions in rabbits and dogs with rabies. These findings were presented in 1903 at a meeting of the Società Medico-Chirurgica of Pavia. Negri was convinced the inclusions were a parasitic protozoon and the etiologic agent of rabies. Later that same year, however, Paul Remlinger and Rifat-Bey Frasheri in Constantinople and, separately, Alfonso di Vestea in Naples showed that the etiologic agent of rabies is a filterable virus. Negri continued until 1909 to try to prove that the intraneuronal neurons named after him corresponded to steps in the developmental cycle of a protozoan. In spite of his incorrect etiologic hypothesis, Negri’s discovery represented a breakthrough in the rapid diagnosis of rabies, and the detection of Negri bodies was used for many years until the development of modern diagnostic methods.
